# The Role and Implications of Epicardial Fat in Coronary Atherosclerotic Disease

**DOI:** 10.3390/jcm11164718

**Published:** 2022-08-12

**Authors:** Laurentiu Braescu, Marinica Gaspar, Darius Buriman, Oana Maria Aburel, Adrian-Petru Merce, Felix Bratosin, Klokov Sergei Aleksandrovich, Satish Alambaram, Cristian Mornos

**Affiliations:** 1 Department of Cardiovascular Surgery, “Victor Babes” University of Medicine and Pharmacy, 300041 Timisoara, Romania; 2 Department of Functional Sciences—Pathophysiology, Faculty of Medicine, “Victor Babes” University of Medicine and Pharmacy, 300041 Timisoara, Romania; 3 Center for Translational Research and Systems Medicine, “Victor Babes” University of Medicine and Pharmacy, 300041 Timisoara, Romania; 4 Methodological and Infectious Diseases Research Center, Department of Infectious Diseases, “Victor Babes” University of Medicine and Pharmacy, 300041 Timisoara, Romania; 5 NV Sklifosovsky Research Institute for Emergency Medicine, Bolshaya Sukharevskaya Ploshchad 3, 129090 Moscow, Russia; 6 Bhaskar Medical College, Amdapur Road 156-162, Hyderabad 500075, India; 7 Discipline of Cardiology, “Victor Babes” University of Medicine and Pharmacy, Eftimie Murgu Sq. No. 2, 300041 Timisoara, Romania

**Keywords:** epicardial fat, atherosclerosis, coronary artery disease, metabolic syndrome, oxidative stress

## Abstract

The current minireview aims to assess the implications of epicardial fat secretory function in the development of coronary artery disease. The epicardial adipose tissue (EAT) is a visceral fat depot that has been described as a cardiovascular risk factor. In addition to its mechanical protection role and physiological secretory function, it seems that various secretion products of the epicardial fat are responsible for metabolic disturbances at the level of the cardiac muscle when in association with pre-existing pathological conditions, such as metabolic syndrome. There is a pathological reduction in sarcomere shortening, abnormal cytosolic Ca^2+^ fluxes, reduced expression of sarcoplasmic endoplasmic reticulum ATPase 2a and decreased insulin-mediated Akt-Ser473-phosphorylation in association with abnormal levels of epicardial fat tissue. Activin A, angiopoietin-2, and CD14-positive monocytes selectively accumulate in the diseased myocardium, resulting in reduced cardiomyocyte contractile function. At the same time, it is believed that these alterations in secretory products directly decrease the myocyte function via molecular changes, thus contributing to the development of coronary disease when certain comorbidities are associated.

## 1. Introduction

The views regarding the function of epicardial adipose tissue (EAT) in heart physiology include its role in cardiac metabolism, the mechanical protection of coronaries [1], innervation, and potentially the cryoprotection of the heart too. Nevertheless, recent evidence has revealed that EAT regulates multiple aspects of cardiac biology, including myocardial redox state, intracellular Ca^2+^ cycling [2], the electrophysiological and contractile properties of cardiomyocytes, and cardiac fibrosis, as well as atherogenesis. Numerous studies discovered that both epicardial and intra-thoracic fat were associated with metabolic syndrome. However, only epicardial fat is an independent risk factor for the accelerated progression of sub-clinical coronary atherosclerosis [3]. As determined by non-contrast computed tomography, epicardial fat volume is a reliable indicator of the existence and severity of coronary calcium load and identifies people at higher risk of coronary artery disease development. Moreover, it is now known that complicated bidirectional pathways control the connection between EAT and heart function because not only do the adipokines influence cardiac function, but also the heart impacts EAT biology via a paracrine ‘reverse’ signaling [4]. Such complex interactions, as well as epicardial fat accumulation and epicardium to adipocyte differentiation, should be considered by clinical studies investigating EAT as a risk marker and its potential as a therapeutic target in cardiovascular disease. Further in-depth study of the molecular processes controlling the cross-talk between the heart and EAT is anticipated to improve our knowledge of the function of the latter in cardiac physiology and related disease mechanisms. Recent studies [5] observed a pathological reduction in sarcomere shortening, abnormal cytosolic Ca^2+^ fluxes, the reduced expression of sarcoplasmic endoplasmic reticulum ATPase 2a, and decreased insulin-mediated Akt-Ser473-phosphorylation in association with abnormal levels of epicardial fat tissue. Activin A, angiopoietin-2, and CD14-positive monocytes selectively accumulate in the diseased myocardium, resulting in reduced cardiomyocyte contractile function.

This minireview is aimed to (1) summarize anatomical, physiological, and pathophysiological characteristics of EAT and (2) briefly review non-invasive methods for both qualitative and quantitative evaluation of epicardial fat. The premise of this work is a better understanding of epicardial fat secretory function, as it is believed it can explain some particularities in the progression of coronary artery disease (CAD), especially in association with metabolic syndrome [6,7].

## 2. Anatomy

**Figure 1:**
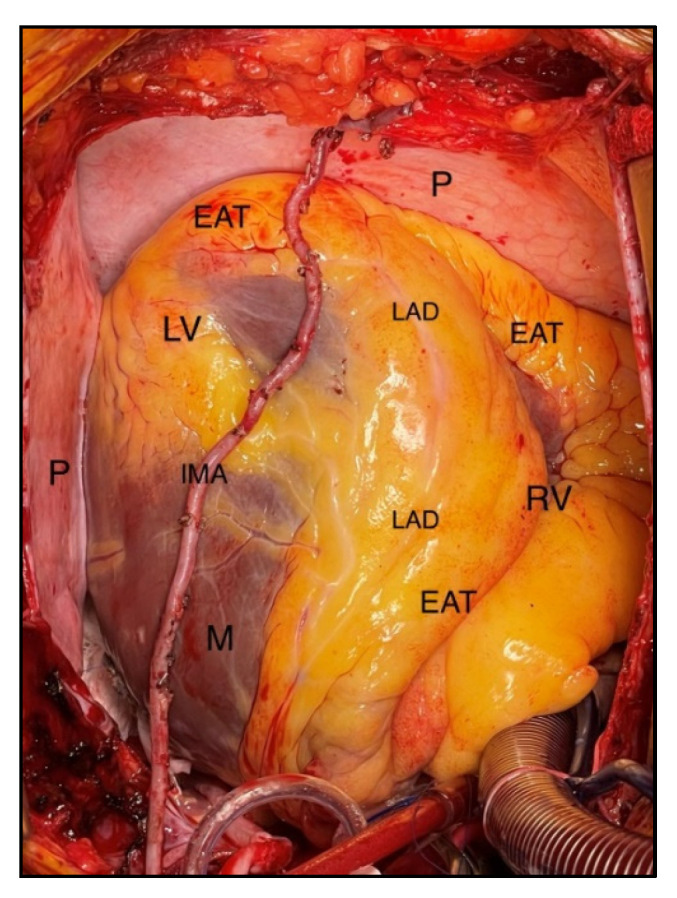
Intra-operative view of EAT. Left ventricle (LV); Right ventricle (RV); Left anterior descending artery (LAD); Internal mammary artery (IMA); Epicardial adipose tissue (EAT); Myocardium (M); Pericardium (P). Photo collected from own practice and obtained with patient’s consent.

The adipose tissue of the heart can be separated into two different compartments by their proximity to the heart muscle, as epicardial fat and pericardial fat. The epicardial fat is located between the visceral pericardium and the myocardium, while the pericardial fat is located on the outer surface of the parietal pericardium [1,2,3,4,5,6], as seen in Figure 1. In addition to these two layers of fatty tissue, anatomical data from many histological species indicate that fatty infiltrate is also present at the level of the myocardium, although the fat distribution in the atrial and ventricular myocardium is extremely diverse. In particular, atrial appendages have fat deposits, particularly around the left posterior atrium [8]. Whereas such fat distributions are deemed normal, fatty infiltration is considered arrhythmogenic, and a variety of pathological cardiac disorders are associated with increased myocardial fat deposits, particularly in the epicardium [9]. Although numerous hypotheses have been advanced about the physiological and pathological functions of epicardial fat, this subject remains poorly known.

Epicardial and pericardial fat is different in many ways, including structure, origin, and vascularization. Epicardial fat originates from splanchnopleuric mesoderm, while the pericardial fat originates from the primitive thoracic mesenchyme [1,2,3,4]. As for vascularization, epicardial fat is supplied by coronary arteries, while pericardial fat is supplied by branches of the internal thoracic artery [1,2,3,4,7]. The embryological origin of epicardial fat is the same as for omental and mesenteric adipose tissues [7].

In adults, EAT is more commonly found in the atrioventricular and interventricular grooves and right ventricular free wall but may also be located on atria surrounding the coronary arteries and the apex of the left ventricle [10]. According to several articles [11,12,13], in adults, physiological epicardial fat represents approximately 20% of the heart’s mass.

**Figure 2:**
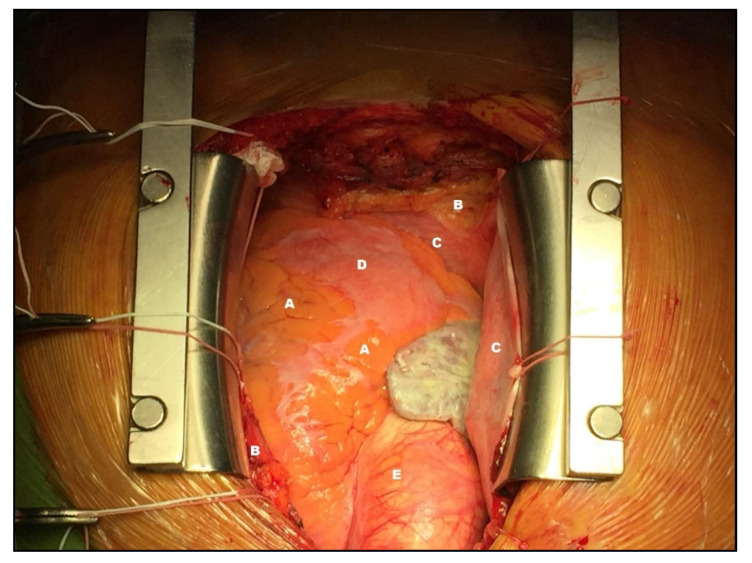
Anatomical description. Epicardial fat (A). Pericardial fat (B). Pericardium (C). The myocardium (D). Aorta (E). Photo collected from own practice and obtained with patient’s consent.

An anatomical distinction may be made in describing epicardial fat [14], namely peri-coronary epicardial and peri-myocardial EAT, as seen in Figure 2. The distinction is due to the location of the fat that, in the first category, is located around the coronary arteries, while the myocardial epicardial fat is located at a distance of about 10–20 mm from the coronary arteries directly in the myocardium. It is indicated that cardiac illness might result due to an increase in EAT, which is linked with arrhythmogenicity [15]. It has been hypothesized that when EAT increases, excess adiposity, cytokines, free fatty acids, and other bioactive chemicals concentrate in the proximity of myocytes, posing structural and electrical challenges. These remodeling mechanisms affect the myocardium’s normal impulse initiation and propagation characteristics [15].

Adipocytes are the largest part of the epicardial fat composition that are generally smaller in size than those in subcutaneous cellular tissue and pericardial fat. In addition to adipocytes, the epicardial also contains ganglia, interconnecting nerves, resident monocytes, and immune cells [11,16].

## 3. Physiology and Pathophysiology

### 3.1. The Various Roles of Epicardial Fat

The high energy demand of the myocardium is achieved by beta-oxidation of long-chain fatty acids. Between 50–70% of the energy production of the heart is achieved by the metabolism of free fatty acids (FFAs), which is accomplished by the myocardium [17]. The close correlation between the volume of epicardial fat and the concentration of triglycerides (converted from free fatty acids in the blood) in the myocardium has already been demonstrated [18]. Major differences between epicardial fat and non-EAT have been described. The higher capacity of releasing FFAs represents the greater difference between epicardial fat and other visceral adipose tissue elsewhere in the body [19]. Compared to perirenal depots of adipose tissue, epicardial fat has twice as high a release rate of fatty acids and the maximum FFA synthesis rate, which is significantly higher. Another difference from intra-abdominal depots of adipose tissue is that epicardial fat has a very low ability to use glucose [20]. In contrast to subcutaneous fat depots, epicardial fat has consistently reduced lipoprotein lipase and acetyl-CoA carboxylase activity. Compared to other visceral adipose tissue, epicardial fat contains more proteins, while mitochondrial content is almost similar [1]. Mazurek et al. showed that the epicardial fat obtained during myocardial revascularization by aortocoronary bypass contains a dense inflammatory cell infiltration, largely represented by macrophages [16]. Another research found that the relationship between coronary heart disease and epicardial adipose tissue is not significantly correlated with the obesity status of the patient. [11].

In the epicardial fat, the saturated fatty acids, including myristic acid (14:0), palmitic acid (16:0), and stearic acid (18:0), are better represented, and the unsaturated ones, including palmitoleic acid (16:1n−7), oleic acid (18:1n−9), linoleic acid (18:2n−6), and linolenic acid (18:3n−3) are less represented than the subcutaneous fat [20,21].

The coronary arteries and the entire myocardium may thereby be affected by this active metabolic organ (i.e., the epicardial fat), which is an important source of bioactive molecules: adiponectin, resistin, and inflammatory cytokines. The role of adiponectin in decreasing FFA circulating levels and enhancing insulin sensitivity is well known, while resistin contribution to atherogenesis by smooth muscle cells and vascular endothelial cells is still a subject of research [1,16]. There are two pathways by which epicardial fat and myocardium interact: paracrine and vasocrine, the latter being defined as the mechanism linking insulin resistance to vascular disease. The wall of coronary arteries can be traversed by diffusion from the outside to the inside following the paracrine release of cytokines from endothelial cells, while adipokines and FFA, via a vasocrine mechanism, can be released from the epicardial tissue into the vasa vasorum and then transported to the arterial wall [3].

Oxidative stress from epicardial fat has been described as being higher than in subcutaneous fat, involving vasoactive factors and cytokines. Leptin, angiotensin II and nitric oxide have a clearly established relation. Angiotensin II induces contractility in the vascular smooth muscle cells of the aorta, while leptin inhibits its vasoconstrictive effect by a nitric oxide-dependent mechanism [22]. However, the role of these epicardial adipose tissue factors in the endothelium of the coronary arteries is not yet well known. Moreover, abnormal glucose metabolism is associated with atherogenesis [23]. Epicardial fat can also contribute to atherosclerosis by mechanical effects, such as the attenuation of artery torsion under physiological conditions, while excessive amounts of fat around the coronary arteries may be harmful. Asymmetric vascular remodeling is due to the presence of atherosclerotic plaques. An easier expansion of the vessel wall is related to coronary lesions that are surrounded by epicardial fat to those surrounded by myocardium due to extravascular resistance [24].

According to several studies, epicardial fat can have a protective effect due to the presence of anti-inflammatory and anti-atherogenic adipokines, such as adiponectin and adrenomedullin, which is a powerful vasodilator and an antioxidative peptide. In the case of adiponectin, its serum expression is significantly lower in patients with coronary artery disease (CAD). After myocardial revascularization by aortocoronary bypass and elevated hemodynamic conditions, increased levels of adrenomedullin have also been reported [12,25].

Physiological roles of epicardial fat include an indispensable energy source for the myocardium, protective effects for both myocardium and coronary arteries from inflammatory cells, as well as a source of adipokine with an anti-atherogenic role in the early stages of atherosclerosis, thus providing mechanical protection for the coronary arteries by extending the arterial wall [11,13]. FFAs express pathophysiological roles of excessive epicardial fat, representing an important source of proinflammatory and proatherogenic adipokines and explaining the correlation of pathological EAT with atrial fibrillation, CAD, and ventricular hypertrophy [26,27].

### 3.2. Epicardial Fat in Relationship with CAD

**Figure 3:**
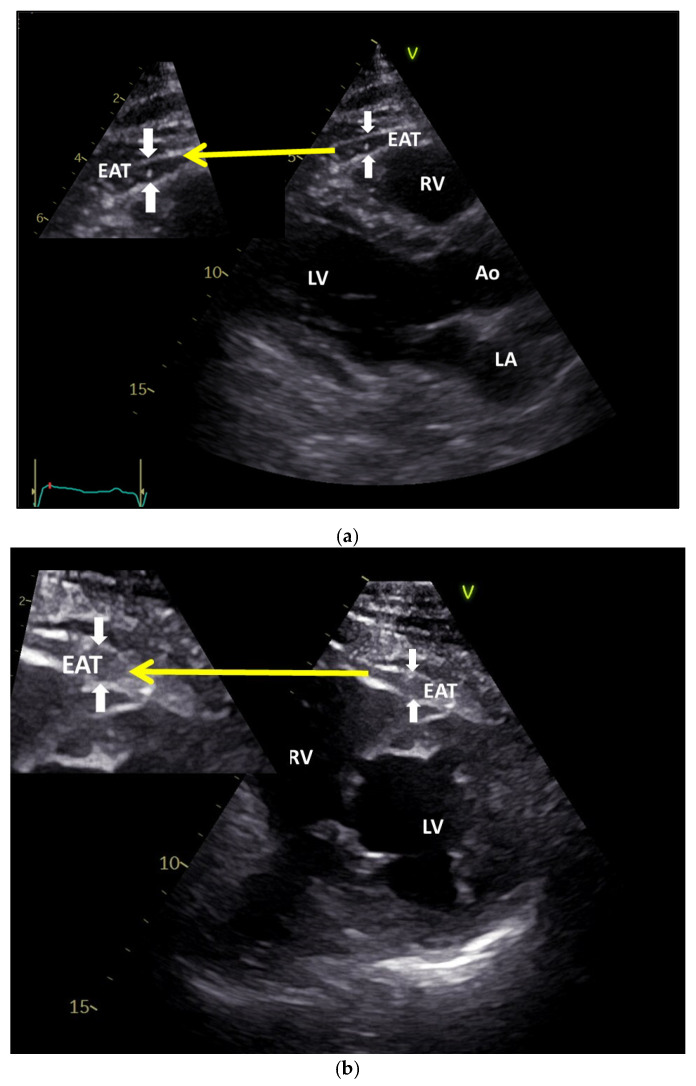
Epicardial fat by transthoracic echocardiography. (**a**). Epicardial fat thickness at parasternal long axis (the distance from outer side of myocardium to pericardium). (**b**). Epicardial fat by transthoracic echocardiography. Epicardial fat thickness at parasternal short axis (the distance from outer side of myocardium to pericardium). EAT—Epicardial Adipose Tissue; RV—Right Ventricle; LV—Left Ventricle; Ao—Aorta; LA—Left Atrium. White arrows pointing towards the EAT; Yellow arrow pointing towards the magnified view of EAT.

CAD can be influenced by the presence of epicardial fat due to its paracrine effects, causing the development and progression of coronary plaques. Eroglu et al. showed in a study of 150 participants that the thickness of EAT was significantly higher (6.9 ± 1.5 mm) in patients with CAD compared to those with normal coronary arteries (4.4 ± 0.8 mm), being easily and non-invasively assessed using transthoracic ultrasound [28], as seen in Figure 3a,b, and confirmed in a series of studies on the ultrasound findings of EAT [29,30,31,32,33]. It has been demonstrated using the CT investigation method, that the epicardial fat volume is significantly higher in patients presenting CAD (≈151.9 cm³) than those with normal coronary arteries (≈74.8 cm³), and this is directly associated with the high-risk coronary lesion morphology [34,35].

It has been shown that epicardial fat is a source of inflammatory mediators. During myocardial revascularization by aortocoronary bypass, epicardial fat tissue and subcutaneous tissue were harvested in order to compare the expression of inflammatory mediators. The presence of inflammatory cytokines (interleukin-1β, interleukin-6, tumor necrosis factor (TNF), and monocyte chemotactic protein (MCP) were significantly higher in epicardial fat deposits, compared to the subcutaneous ones [16]. Similarly, other studies highlighted the role of the macrophages in EAT inflammation, as Baker et al. stated in their study that in CAD patients, there is an increase in the expression of the nuclear factor kappaB (NF-kB) and c-Jun N-terminal kinase (JNK), compared to patients without CAD; also between CD-68 and toll-like receptor-2 and toll-like receptor-4 and TNF-α, a strong correlation has been demonstrated [36]. Furthermore, in patients with CAD, the expression of protective mediators (adiponectin and adrenomedullin) from epicardial fat is much lower than in those with normal coronary arteries [12,25,37]. Protective adipokines, such as adiponectin, adrenomedullin, and apelin have a beneficial effect due to anti-inflammatory, anti-hypertrophic, cardioprotective, and vasodilatory effects.

A recent cross-sectional study investigated the EAT volume and coronary calcification among 409 patients living with diabetes [38]. The average BMI of the study participants was 29 kg per square meter, and the mean epicardial fat volume was 93 cubic centimeters. EAT volume was positively associated with age, BMI, pack-year history of smoking, and triglyceridemia but negatively correlated with HDL cholesterol level. Additionally, it was lower in persons with retinopathy, but higher in males, people who are Caucasian, patients on antihypertensive and lipid-lowering medicine, individuals with nephropathy, and lastly, those with coronary artery calcification (CAC) levels greater than 100 Agatston Units (AU). Conclusively, the EAT volume, age, type 2 diabetes mellitus, ethnicity, antihypertensive and lipid-lowering medicine, cumulative cigarette use, retinopathy, retinal edema, and macrovascular disease were all associated with CAC 100 AU. Moreover, the multivariable analysis adjusting for all of these variables, as well as gender and BMI revealed that EAT volume was independently related to CAC > 100 AU [38].

### 3.3. Non-Invasive Measurement of Epicardial Fat: Ultrasound, Computed Tomography (CT), and Magnetic Resonance Imaging (MRI)

To evaluate the thickness and volume of epicardial fat, several non-invasive methods are used in clinical practice. Transthoracic echocardiography is the main tool used to quantify and measure the thickness and the volume of epicardial fat [39]. Computer tomography (CT) and magnetic resonance imaging (MRI) are mainly used to evaluate the volume of EAT because of their high spatial resolution [40].

Using transthoracic two-dimensional (2D) echocardiography, epicardial fat is visualized as an echo-free space between the visceral pericardium and the outer wall of the myocardium. The measurements are usually made on the free wall of the right ventricle because here, the thickness of the adipose tissue is greatest and can be seen both in the long and short parasternal axis [12]. In a study of 246 patients, 58% with metabolic syndrome, Iacobellis et al., using echocardiography, showed that the median values of epicardial fat thickness of 9.5 mm in males and 7.5 mm in females were significantly higher than in patients without metabolic syndrome. These values were considered threshold values for high-risk echocardiographic epicardial fat thickness, and this measurement may be helpful in assessing cardiometabolic risk [27,41]. Three-dimensional echocardiography can help with a better assessment of the epicardial fat volume.

Coronary computed tomography angiography (CTA) has become one of the most important diagnostic imaging modalities for the evaluation of coronary artery diseases. During coronary computed CTA, sufficient vascular enhancement is essential for the accurate detection and evaluation of lesions in the coronary arteries [6,42]. Computer tomography (CT) can accurately measure the thickness, volume, and total area of the fat that surrounds the heart. The thickness of epicardial fat can be measured, similar to echocardiography, on the right ventricular free wall or in the inter- and atrioventricular grooves. At the level of the three main coronary arteries (anterior descending artery, circumflex artery, and right coronary artery), EAT is measured in an axial view, perpendicular to the surface of the heart [31]. According to a study that included 358 patients who had acute chest pain and who underwent CT angiography, the volume of epicardial fat was evaluated based on the coronary lesions they presented. Thus, in patients with a high-risk coronary lesion, the EAT was found to be twice as high as those without CAD [32].

Although CT still remains the traditional gold standard for evaluating EAT [43], magnetic resonance imaging (MRI) can be considered an emerging gold standard due to the high resolution of the obtained images. As well as in the CT evaluation, epicardial adipose tissue contours are traced, and adipose tissue voxels are added to calculate the epicardial fat volume [33].

## 4. Conclusions

The current study gathers a comprehensive review of literature on the pathophysiology of EAT in correlation with imaging and intra-operative findings from personal experience. EAT is a fat depot that surrounds the heart with unique anatomical and functional features, and it is consistently correlated with metabolic syndrome and CAD. Recent studies successfully demonstrated the direct relationship between epicardial fat volume and histological findings with the presence of CAD using imaging methods [44]. Simple and reproducible using ECG-gated cardiac CT without contrast injection or transthoracic ultrasound are very useful tools for the primary assessment methods to establish the possible correlation between the volume of epicardial fat and CAD.

## Data Availability

Not applicable.
